# Characterization of flow‐regulated cortical collecting duct endothelin‐1 production

**DOI:** 10.14814/phy2.13126

**Published:** 2017-02-27

**Authors:** Nirupama Ramkumar, Yang Gao, Donald E. Kohan

**Affiliations:** ^1^ Division of NephrologyUniversity of Utah Health Sciences CenterSalt Lake CityUtah; ^2^ Salt Lake VA Medical CenterSalt Lake CityUtah

**Keywords:** Collecting duct, cortical, endothelin, flow, medullary, signaling

## Abstract

Collecting duct (CD) endothelin‐1 (ET‐1) is an autocrine inhibitor of Na^+^ and water reabsorption. Salt or water loading increases CD ET‐1 production; this is likely due, at least in part, to increased tubule fluid flow. The mechanisms by which flow stimulates CD ET‐1 production are incompletely understood. In particular, flow induction of cortical CD (CCD) and inner medullary CD (IMCD) ET‐1 synthesis may occur via different mechanisms. Since flow‐mediated ET‐1 production in IMCD has been more extensively characterized than in the CCD, this study was undertaken to further examine putative signaling pathways involved in flow‐stimulated CCD ET‐1 production. The CD cell line, mpkCCDcl4, was exposed to static or flow (2 dyne/cm^2^ for 2 h) conditions and ET‐1/GAPDH mRNA levels were assessed. Intracellular Ca^2+^, Ca^2+^‐stimulated Ca^2+^ release, calcineurin, and protein kinase c *α*/*β* isoforms were all involved in the ET‐1 flow response. TRPC6, but not other CD‐expressed TRP channels (TRPC3, 4, and 5, or TRPV4) played a role in the ET‐1 flow response. Purinergic signaling pathways and cilia were not involved in the ET‐1 flow response. Based on these and previously published findings, we present a comparison of flow‐stimulated CD ET‐1 production between CCD and IMCD. We suggest that flow‐stimulated CCD ET‐1 production may be more involved in responding to Na^+^ delivery, while IMCD ET‐1 production may be more responsive to water and solute delivery; the responsible pathways for mediating these effects in the two regions of the CD appear to be substantially distinct from one another.

## Introduction

Collecting duct (CD)‐derived endothelin‐1 (ET‐1) is an important regulator of renal Na^+^ and water excretion, and blood pressure. The CD may produce more ET‐1 than any other cell type in the body (Kohan 1991; Kohan et al. 2011). ET‐1 reduces Na^+^ and water reabsorption in the CD via inhibition of the epithelial Na^+^ channel (ENaC), the Na^+^/K^+^ ATPase, and vasopressin (AVP)‐stimulated adenylyl cyclase activity (Tomita et al. 1990, 1993; Bugaj et al. 2008; Pavlov et al. 2010). CD‐specific knockout of ET‐1 causes renal Na^+^ and water retention, and hypertension (Ahn et al. 2004; Ge et al. 2005). Hence, CD‐derived ET‐1 exerts natriuretic, diuretic, and antihypertensive effects under physiological conditions.

CD ET‐1 production and urinary ET‐1 excretion are increased by body fluid volume expansion in experimental animals and humans (Malatino et al. 2000; Cuzzola et al. 2001; Mawji et al. 2004). The mechanisms by which this occurs have been partially elucidated. In general, circulating hormones do not appear to be responsible for augmenting CD ET‐1 production in response to body volume expansion (Kohan et al. 2011); rather, local factors may be of primary importance. In particular, tubule fluid flow has been demonstrated to increase CD ET‐1 production; this occurs in both cortical CD (CCD) (Lyon‐Roberts et al. 2011) and inner medullary CD (IMCD) (Pandit et al. 2015). However, the mechanisms/signaling pathways by which this occurs may differ between the two CD regions. To date, no direct comparison of the pathways by which flow enhances regional CD ET‐1 production has been conducted. Consequently, we herein examine and compare mechanisms by which CCD and IMCD ET‐1 production is increased in response to flow. Because the majority of flow‐induced ET‐1 production pathway analysis has been conducted in IMCD cells, this study focused on CCD cells.

## Materials and Methods

### Materials

Calphostin C, Pyr3, and SKF‐96365 were obtained from Tocris Bioscience (Ellisville, MD). All other reagents were obtained from Sigma Chemical Co. (St. Louis, MO) unless specifically stated otherwise.

### Cell culture

The CCD cell line, mpkCCDc14 (MPK‐CCD), was provided by Dr. Alain Vandewalle at INSERM, France (Bens et al. 1999). Cells were grown to confluence at 37°C in 5% CO_2_ in 50:50 DMEM:F12 containing 2 *μ*g/mL dexamethasone, insulin, transferrin, selenium, 400 nmol/L triiodothyronine, 1 *μ*g/mL epidermal growth factor, 2 mmol/L glutamine, 1 mg/mL penicillin, 1 mg/mL streptomycin, and 2% fetal bovine serum.

### Flow studies

Confluent MPK‐CCD cells, grown on 10‐cm dishes, were rinsed with Hanks balanced salt solution (HBSS) and chambers attached to the plates as previously described (Lyon‐Roberts et al. 2011). In brief, a rectangular parallel plate flow chamber (Cat. No. 31‐010, Glycotech, Gaithersburg, MD) was vacuum sealed onto a portion of the 10‐cm dish using a silastic gasket that runs around the periphery of the chamber; the plate has two manifolds through which medium enters and exits the channel. A pump (Ismatec, Glattbrugg, Switzerland) drives the recirculating perfusate (~5 mL). Flow rate and time were set at the optimal ET‐1 mRNA induction conditions (2 dyne/cm^2^ for 2 h) as determined in previous studies (Lyon‐Roberts et al. 2011; Pandit et al. 2012, 2015). Perfusion fluid were HBSS + 10 mmol/L HEPES (pH 7.4). All experiments were conducted at 37°C. RNA was isolated from the cells exposed to perfusion as described below.

### ET‐1 mRNA

RNA was extracted using a RNeasy mini kit (Qiagen, Germantown, MD), reverse transcribed, and cDNA levels for ET‐1 and GAPDH determined using real‐time PCR (StepOnePlus, Applied Biosystems, Foster City, CA). PCR was performed according to the manufacturer's instructions using the Taqman Gene Expression Assay (Applied Biosystems) with ET‐1 (Cat. No. Mm00438656_m1) and GAPDH (Cat. No. Mm03302249_g1) primers.

### Statistics

Data are presented as mean ± SE. Differences between groups were determined using two‐way analysis of variance with the post hoc Scheffe test. A value of *P* < 0.05 was taken as significant.

## Results

### Role of intracellular Ca^2+^‐dependent signaling pathways in static and flow‐induced CCD ET‐1 production

Previous studies in mouse IMCD (IMCD3) cells showed that flow‐stimulated ET‐1 production was dependent upon PKC and Ca^2+^‐signaling pathways (Pandit et al. 2015); however, these pathways have not been well analyzed in CCD. Consequently, several pharmacologic tools were used to assess the ET‐1 flow response in mouse CCD cells (MPK‐CCD). For these and all subsequent experiments reported herein, cells were exposed to static or flow (2 dyne/cm^2^) conditions for 2 h; these flow conditions were shown to optimally induce ET‐1 production and were used in all previous studies with IMCD3 and MPK‐CCD cells examining the ET‐1 flow response (Lyon‐Roberts et al. 2011; Pandit et al. 2015). It is also important to note that ET‐1 mRNA is assessed instead of ET‐1 protein. As previously reported (Lyon‐Roberts et al. 2011; Pandit et al. 2012, 2015), ET‐1 protein levels are too low to detect in the small amount of cells exposed to flow; however, ET‐1 mRNA, which has a very short half‐life (~15 min) has been shown to accurately reflect ET‐1 protein levels (Kohan et al. 2011).

**Figure 1:**
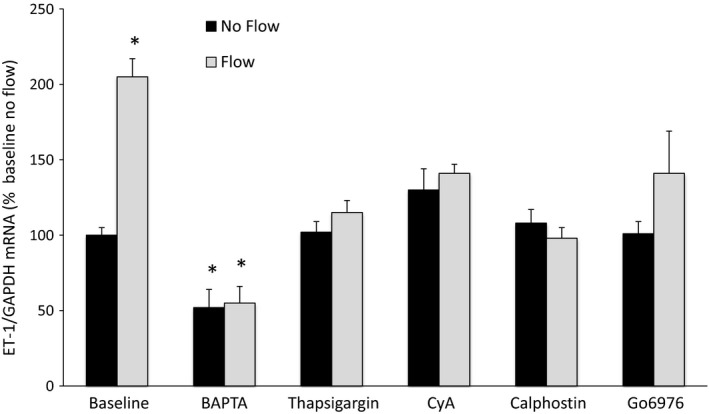
Role of Ca^2+^ signaling pathways in flow‐stimulated endothelin‐1 (ET‐1) mRNA levels in MPK‐CCD cells. Cells were preincubated with vehicle, 50 *μ*mol/L BAPTA‐AM, 200 nmol/L thapsigargin, 3 *μ*g/mL cyclosporine (CyA), 0.1 *μ*mol/L calphostin C, or 200 nmol/L Go6976 for 30 min, followed by exposure to static conditions or shear stress at 2 dyne/cm^2^ for 2 h in the presence of the same agents, then determination of ET‐1/GAPDH mRNA levels. *N* = 8–12 each data point. *P < 0.05 versus baseline (vehicle alone) no flow conditions.

As previously reported (Lyon‐Roberts et al. 2011), BAPTA‐AM (the cell permeable intracellular Ca^2+^ chelator) and calphostin C (PKC inhibitor) blocked flow‐stimulated ET‐1 production (Fig. 1). In addition, BAPTA also reduced ET‐1 mRNA levels in cells not exposed to flow (static conditions). Inhibition of Ca^2+^‐stimulated Ca^2+^ release with thapsigargin and calcineurin antagonism with cyclosporine A also abolished the ET‐1 flow response. Finally, inhibition of PKC‐*α* and ‐*β* isoforms with Go6976 abolished the ET‐1 flow response.

### Role of Ca^2+^channels in static and flow‐induced CCD ET‐1 production

**Figure 2:**
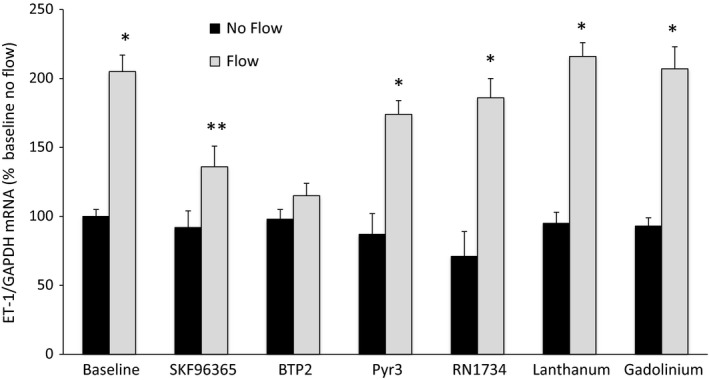
Role of Ca^2+^ channels in flow‐stimulated endothelin‐1 (ET‐1) mRNA levels in MPK‐CCD cells. Cells were preincubated with vehicle, 20 *μ*mol/L SKF96265, 3 *μ*mol/L BTP2, 10 *μ*mol/L Pyr3, 30 *μ*mol/L RN1734, 10 *μ*mol/L lanthanum, or 10 *μ*mol/L gadolinium for 30 min, followed by exposure to static conditions or shear stress at 2 dyne/cm^2^ for 2 h in the presence of the same agents, then determination of ET‐1/GAPDH mRNA levels. *N* = 8–12 each data point. *P < 0.05 versus baseline (vehicle alone) no flow conditions; **P < 0.05 versus baseline no flow conditions and versus baseline flow conditions.

Previous studies in IMCD cells demonstrated no role for IMCD‐expressed TRPC and TRPV channels reported to be expressed in this cell type (Pandit et al. 2015). To assess the role of these Ca^2+^channels in the ET‐1 flow response, a variety of pharmacologic inhibitors of Ca^2+^channels expressed in CD were utilized (Fig. 2). Inhibition of TRPC6 with SKF96365 markedly reduced flow‐stimulated ET‐1 production, while BTP2, which inhibits both TRPC3 and TRPC6, completely abolished the ET‐1 flow response. A role for TRPC3 per se in the ET‐1 flow response was not supported by the finding that Pyr3, a specific TRPC3 antagonist, had no effect on flow‐induced ET‐1 mRNA levels. Neither lanthanum nor gadolinium, which both stimulate TRPC4 and TRPC5 activity, altered ET‐1 production under static or flow conditions. Finally, RN1734, which blocks TRPV4, had no effect on the ET‐1 flow response. Taken together, these data suggest that TRPC6, but not TRPC3‐5, or TRPV4, mediate, at least in part, the ET‐1 flow response.

### Role of cilia in static and flow‐induced CCD ET‐1 production

**Figure 3:**
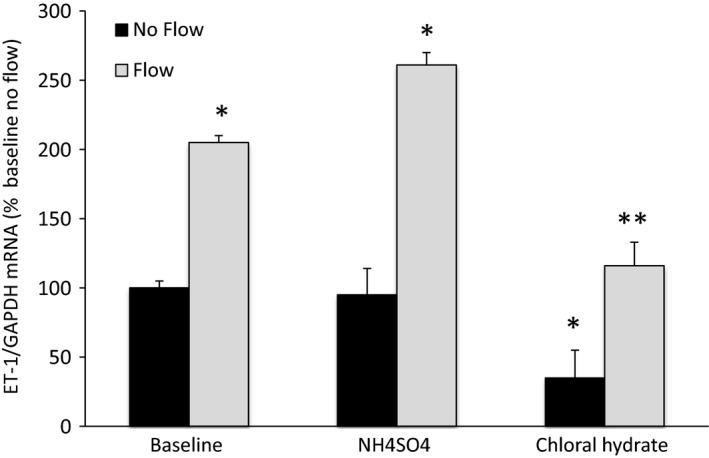
Role of agents that remove cilia on flow‐stimulated endothelin‐1 (ET‐1) mRNA levels in MPK‐CCD cells. Cells were preincubated for 3 h with vehicle or 30 mmol/L NH_4_ SO_4_, or with vehicle or 4 mmol/L chloral hydrate for 24 h, followed by exposure to static conditions or shear stress at 2 dyne/cm^2^ for 2 h in the presence of the same agents, then determination of ET‐1/GAPDH mRNA levels. *N* = 8–12 each data point. *P < 0.05 versus baseline (vehicle alone) no flow conditions; **P < 0.05 versus chloral hydrate no flow conditions.

Previous studies in IMCD3 cells suggested that cilia or proteins that can be associated with cilia (e.g., polycystin 2) are involved in the ET‐1 flow response (Pandit et al. 2015). We initially tried siRNA knockdown to examine the role of polycystins in MPK‐CCD ET‐1 flow responses, however, we were unable to successfully transfect these cells and have them stay attached to the plate during flow. This was most likely due to having to transfect subconfluent MPK‐CCD cells – the cells did not rapidly grow after transfection and were not confluent during the times (2–3 days) after transfection when one optimally detects the effect of the knockdown. Consequently, cells were treated with either NH_4_SO_4_ or chloral hydrate under conditions previously shown to remove cilia (Fig. 3) (Pandit et al. 2015); removal of cilia was confirmed by immunostaining for acetylated *α*‐tubulin (data not shown). Exposure to NH_4_SO_4_ had no effect on static or flow‐stimulated ET‐1 production. Chloral hydrate reduced both static and flow‐induced ET‐1 production by about 60%; however, flow increased ET‐1 production to the same degree as in vehicle‐treated cells. Hence, no evidence was detected that cilia are involved in the CCD ET‐1 flow response.

### Role of the purinergic system in static and flow‐induced CCD ET‐1 production

**Figure 4:**
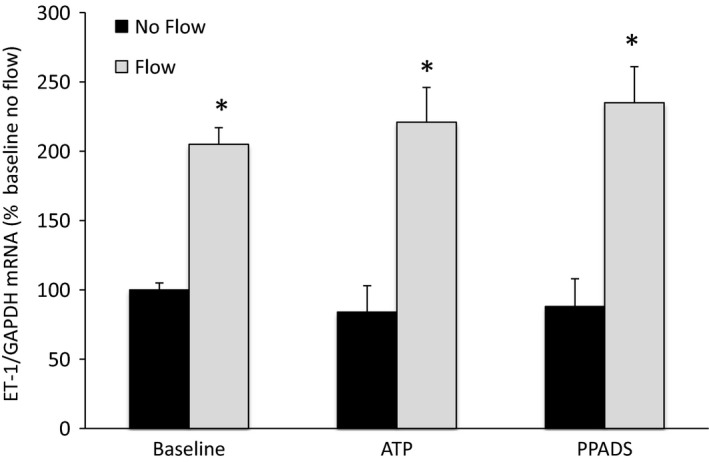
Role of purinergic agents on flow‐stimulated endothelin‐1 (ET‐1) mRNA levels in MPK‐CCD cells. Cells were preincubated with vehicle, 30 *μ*mol/L ATPγS, or 30 *μ*mol/L PPADS for 30 min, followed by exposure to static conditions or shear stress at 2 dyne/cm^2^ for 2 h in the presence of the same agents, then determination of ET‐1/GAPDH mRNA levels. *N* = 8–12 each data point. *P < 0.05 versus baseline (vehicle alone) no flow conditions.

Previous studies in IMCD cells indicated that the purinergic system is an important mediator of flow‐induced ET‐1 production, involving both P2X and P2Y receptors (Pandit et al. 2015). Consequently, the effect of general inhibition or activation of this system was examined in MPK‐CCD cells (Fig. 4). Incubation with the nonspecific purinergic receptor antagonist, PPADS, had no effect on static or flow‐stimulated MPK‐CCD cell ET‐1 production. Similarly, exposure to the stable ATP analogue (ATP*γ*S) did not alter static or flow‐induced MPK‐CCD ET‐1 production. Hence, ET‐1 production, either static or flow‐stimulated, in MPK‐CCD cells does not appear to involve purinergic signaling.

## Discussion

This study demonstrates that flow‐stimulated ET‐1 production by CCD cells: (1) is dependent upon signaling pathways that involve intracellular Ca^2+^, calcineurin, release of Ca^2+^ from intracellular stores, and protein kinase C‐*α* and/or ‐*β* isoforms; (2) depends upon TRPC6, but not TRPC3‐5, or TRPV4, channels; (3) does not involve purinergic receptors; and (4) may not require cilia.

The current studies confirm that flow‐stimulated ET‐1 production in the CCD requires intracellular Ca^2+^ and PKC, similar to the requirements for these factors in the IMCD ET‐1 flow response (Pandit et al. 2015). The current studies also demonstrate a requirement for TRPC6 in the CCD ET‐1 flow response; however, the precise role that TRPC6 plays, and how TRPC6 is activated by flow, were not determined in the current study. Nonetheless, it seems likely that TRPC6 is involved in aspects of Ca^2+^ signaling. TRPC6 is expressed throughout the CD and is located both apically and basolaterally in CD principal cells (Goel et al. 2006). TRPC6 can be activated by diacylglyerols in kidney epithelial cells (such as would be produced by PKC) and this can increase Ca^2+^ influx and intracellular Ca^2+^ concentration, at least in cells with Ca^2+^‐activated K^+^ currents such as CCD (Estacion et al. 2006). Although we found that thapsigargin reduced the ET‐1 flow response, it is unlikely that TRPC6 plays a role in store‐operated Ca^2+^ channel responses in that STIM1, a key regulator of store‐operated Ca^2+^ channels, does not bind to TRPC6 (Worley et al. 2007). These findings in CCD cells contrast with those previously reported by us in IMCD3 cells wherein TRPC6 was not involved in the ET‐1 flow response (Pandit et al. 2015).

No effect of chemical removal of cilia was detected on the ET‐1 flow response in CCD cells. Since polycystins can be intimately associated with cilia, it would have been of interest to see whether polycystins were involved in the CCD ET‐1 flow response. However, as described earlier, technical issues precluded assessing polycystin function in CCD cells. It is notable that the ET‐1 flow response in IMCD cells required polycystin‐2 (Pandit et al. 2015), suggesting, although not proving, that in contrast to CCD, cilia may be important in the IMCD flow response.

The current study found no role for purinergic signaling in the ET‐1 flow response in CCD cells. This finding is in contrast to the requirement by IMCD cells for both P2X and P2Y signaling for the ET‐1 flow response (Pandit et al. 2015). Why IMCD, but not CCD, cells require purinergic signaling for flow‐stimulated ET‐1 is speculative. Purinergic receptors are located throughout the CD; differences in CD segment P2X and/or P2Y expression do not clearly explain different responses to flow (Vallon 2008). Differences in ciliary involvement may account for some of this difference since: (1) as discussed above, cilia may play a role in the IMCD, but not the CCD, ET‐1 flow response; (2) ATP release has been linked to ciliary bending (Vallon 2008); and (3) P2X7, which can localize to cilia, is required for the IMCD, but not the CCD, ET‐1 flow response (Pandit et al. 2015).

It is fully realized that interpretation of these studies is speculative and that several caveats must be considered. First, all these experiments were performed in cell culture with the usual caveats of translating the in vitro to the in vivo situation. Unfortunately, this is by necessity since there is not a way, at least to our knowledge, to specifically target the CCD versus IMCD in vivo, nor is it possible to perform these studies in acutely isolated perfused CD. That said, it should be noted that MPK‐CCD cells do express numerous transporters that are found in CCD in vivo and these MPK‐CCD proteins are regulated in a similar manner to that described in vivo (Bens et al. 1999). Second, these studies used an array of pharmacologic agents with potential off‐target effects. In particular, studies designed to remove cilia must be interpreted cautiously as these agents can also affect other cytoskeletal systems; in our opinion, in remains to be determined whether cilia, polycystins, or other cilia‐associated proteins are involved in regulation of the CCD ET‐1 flow response. One way to approach this would be to generate CCD cell lines lacking cilia or cilia‐associated proteins; however, this was beyond the scope of the current study.

One must be careful extrapolating the current findings to draw conclusions about their physiological significance. Nonetheless, it is worth noting that previous studies found that the CCD, but not the IMCD, ET‐1 flow response depended upon ENaC‐mediated Na^+^ entry and activation of mitochondrial Na^+^/Ca^2+^ exchange (Pandit et al. 2012). In contrast, the IMCD, but not the CCD, ET‐1 flow response was augmented by increased solute delivery (Pandit et al. 2016). Thus, one might speculate that, at least in the context of flow regulation, the primary role of CCD ET‐1 is to mitigate increases in ENaC‐mediated Na^+^ reabsorption that occur in the setting of increased tubule Na^+^ delivery, while IMCD ET‐1 production may be primarily stimulated by increases in tubule fluid flow per se as well as increases in tubule fluid solute delivery. It is highly likely that this model is too simplistic, but nonetheless may serve as a working hypothesis for future studies.

## Conflict of Interest

None declared.
